# Association between *DRD2/ANKK1* TaqIA Polymorphism and Susceptibility with Tourette Syndrome: A Meta-Analysis

**DOI:** 10.1371/journal.pone.0131060

**Published:** 2015-06-25

**Authors:** Aihua Yuan, Liang Su, Shunying Yu, Chunbo Li, Tao Yu, Jinhua Sun

**Affiliations:** 1 Department of Genetics, Shanghai Mental Health Center, Shanghai Jiao Tong University School of Medicine, Shanghai, China; 2 Center for Translational Neuromedicine and the Department of Neurology, University of Rochester Medical Center, Rochester, New York, United States of America; 3 Shanghai Key Laboratory of Psychotic Disorders, Shanghai Mental Health Center, Shanghai Jiao Tong University School of Medicine, Shanghai, China; 4 Bio-X Center, Key Laboratory for the Genetics of Developmental and Neuropsychiatric Disorders (Ministry of Education), Shanghai Jiao Tong University, Shanghai, China; 5 Department of Medical Psychology, Shanghai General Hospital, Shanghai Jiao Tong University, Shanghai, China; 6 Department of Child & Adolescent Psychiatry, Shanghai Mental Health Center, Shanghai Jiao Tong University School of Medicine, Shanghai, China; Tulane University Health Sciences Center, UNITED STATES

## Abstract

**Background:**

Genetic factors are important in the pathogenesis of Tourette syndrome (TS). Notably, Dopamine receptor D2 (*DRD2*) gene has been suggested as a possible candidate gene for this disorder. Several studies have demonstrated that *DRD2/ANKK1* TaqIA polymorphism is associated with an increased risk of developing TS. However, past results remain conflicting. We addressed this controversy by performing a meta-analysis of the relationship between *DRD2/ANKK1* TaqIA polymorphism and TS.

**Methods:**

Literature was searched in multiple databases including PUBMED, COCHRANE and WEB OF SCIENCE up to July 2014. The number of the genotypes for *DRD2/ANKK1* TaqIA in the TS and control subjects was extracted and statistical analysis was performed using Review Manager 5.0.16 and Stata 12.0 software. Summary odds ratios (ORs) and 95% confidence intervals (95%CIs) were utilized to calculate the risk of TS with *DRD2/ANKK1* TaqIA. Stratified analysis based on ethnicity was also conducted.

**Results:**

523 patients with TS, 564 controls and 87 probands plus 152 relatives from five published studies were finally involved in this meta-analysis. Combined analysis revealed that the overall ORs for the *DRD2/ANKK1* TaqIA A1 allele were 1.69 (95%CIs = 1.42-2.00) in the fixed-effect model and 1.66 (95%CIs = 1.33-2.08) in the random-effects model. Stratification by ethnicity indicated the TaqIA A1 allele was significantly associated with TS in Caucasians (fixed-effect model: OR=1.75, 95%CI = 1.43-2.16; random-effect model: OR=1.69, 95%CI = 1.25-2.28) and in Asians (OR=1.54, 95%CI = 1.12-2.10). Meta-analysis of the A1A1 vs. A2A2 (homozygous model), A1A2 vs. A2A2 (heterozygous model) and A1A1+A1A2 vs. A2A2 (dominant model) of this polymorphism revealed a significant association with TS in overall populations and Caucasians.

**Conclusions:**

This meta-analysis suggested that the *DRD2/ANKK1* TaqIA polymorphism might contribute to TS susceptibility, especially in Caucasian population. However, further investigation with a larger number of worldwide studies should be conducted to verify the association.

## Introduction

Tourette syndrome (TS) is a childhood-onset neuropsychiatric disorder with an estimated prevalence of 0.1–1% in children and adolescents from 5 to 18 years old [1]. TS is clinically characterized by involuntary motor tics and vocal tics as well as psychiatric comorbidities, such as obsessive-compulsive disorder (OCD) and attention deficit hyperactivity disorder (ADHD) [2–4]. The factors that contribute the pathogenesis of this condition are poorly understood. Twin studies and family-based studies have demonstrated that TS is highly inheritable [5–8]. However, identification of definitive susceptibility genes for TS is difficult, probably due in part to clinical heterogeneity, genetic heterogeneity and multigenic interaction of genes with small effects.

Based on pharmacological findings, it is hypothesized that the dysfunction of dopamine system may cause the change of dopamine level leading to TS [9–11], dopamine system-related genes therefore may be good candidates in the genetic basis of TS. Positive association results between TS and some of these genes involved in dopaminergic neurotransmission have been reported [12–15]. Among these genes, dopamine receptor D2 (*DRD2*) gene, encoding a G protein-coupled receptor located on dopaminergic neurons, is one of the most extensively studied genes in the pathogenesis of TS. Abnormalities of *DRD2* have been reported in the patients with TS. Histological studies showed that the DRD2 receptor density was consistently increased in frontal cortex and striatum in patients with TS when compared to the controls [16, 17]. Gene expression profile analyses using mRNA microarray also supported the above studies. Increased expression of *DRD2* in peripheral blood of patients with TS has been identified, and increased mRNA level of *DRD2* gene correlated with tic severity which became particularly significant when the subjects were un-medicated individuals [18]. Interestingly, evidence from animal study demonstrated that Ningdong granule effectively inhibited the symptoms of the patients with TS by promoting dopamine metabolism, reducing dopamine levels and mRNA expression of *DRD2* in the striatum [15]. In addition, *DRD2* gene maps to human chromosome 11q23.2, which was reported to be positive linkage with TS [19]. The above studies have suggested that *DRD2* is a strong candidate gene for TS.

Several studies have attempted to examine the association between *DRD2* variations and TS. The *DRD2/ANNK1* TaqIA (rs1800497) restriction fragment length polymorphism designated has been widely investigated in TS. In the early 1990s, Comings et al. firstly reported that the A1 allele of the TaqIA polymorphism of the *DRD2* gene was associated with TS [20]. However, family-based studies from Diaz-Anzaldua et al., Nothern et al. and Herzberg et al. failed to verify the association of TaqIA polymorphism in *DRD2* for TS [14, 21, 22]. As TS is a complex, clinically heterogeneous and most likely genetic heterogeneous disease, the inconsistent results may be due to the uncertainties in the phenotypic definition of the disease, and to the different prevalence of susceptibility alleles in the different ethnic populations.

Therefore, in order to increase the statistical power for detecting the association and further evaluate the role of TaqIA polymorphism in *DRD2* gene in susceptibility to TS, we performed a meta-analysis of all eligible case-control and family-based studies using Review Manager and Stata program.

## Methods

### Search strategy

PUBMED, COCHRANE and WEB OF SCIENCE databases were retrieved for the association studies focused on the relationship between the polymorphisms of *DRD2* and the risk of TS (the last search update was July 2014). The key words and subject terms used were as follows: “*D2 Dopamine Receptor* or *DRD2*”; “Tourette syndrome or TS or Gilles de la Tourette syndrome”; and “polymorphism or variant”. The research was limited to English-language journals, and the additional studies were identified by a manual search of the reference list from the retrieved studies.

### Inclusion and exclusion criteria

The following criteria were performed for the inclusion of the studies in our meta-analysis: (1) evaluating the relationship of TaqIA polymorphism in *DRD2* gene with TS risk; (2) supplying allele frequency. The major reasons for exclusion of studies were (1) no usable data was reported; (2) the articles were abstracts or reviews, or if they were duplicated. The excluded studies were listed in the S1 Text.

### Data extraction

Data was carefully extracted from all eligible publications independently by two investigators, according to the strict criteria for inclusion. Discrepancies would be discussed and adjudicated by a third investigator until consensus was reached on every item. The following data was extracted from each article: the first author’s name, year of publication, country of origin, ethnicity, diagnostic criteria, study type, genotyping method, number of cases, controls, genotype and allele distributions in cases and controls, and Hardy–Weinberg equilibrium (HWE) of cases and controls.

### Statistical analysis

All analyses were performed on Review Manager 5.0.16 and Stata 12.0. The HWE was used to compare the observed genotype frequencies with expected genotype frequencies in controls. The strength of the association between *DRD2* TaqIA polymorphism and risk of TS was measured by odds ratios (ORs) with 95% confidence intervals (CIs). Heterogeneity among studies was estimated by the I^2^ index and confidence interval around I^2^ [23–24]. If I^2^ test showed I^2^ > 50% and also the 95% confidence interval didn’t include 0, indicating significant heterogeneity, the random-effects model (the DerSimonian and Laird method) was conducted; otherwise the fixed-effects model (the Kazeem–Farrall method) was used [25, 26]. However, due to the small number of studies involved in this meta-analysis, both the random-effects model and the fixed-effects model were applied to all genetic models analysis. Subgroup analysis was conducted with respect to ethnicity. Sensitivity analysis was performed to evaluate the effect of each individual study on the pooled ORs.

## Results

### Literature selection and study characteristics

**Fig 1:**
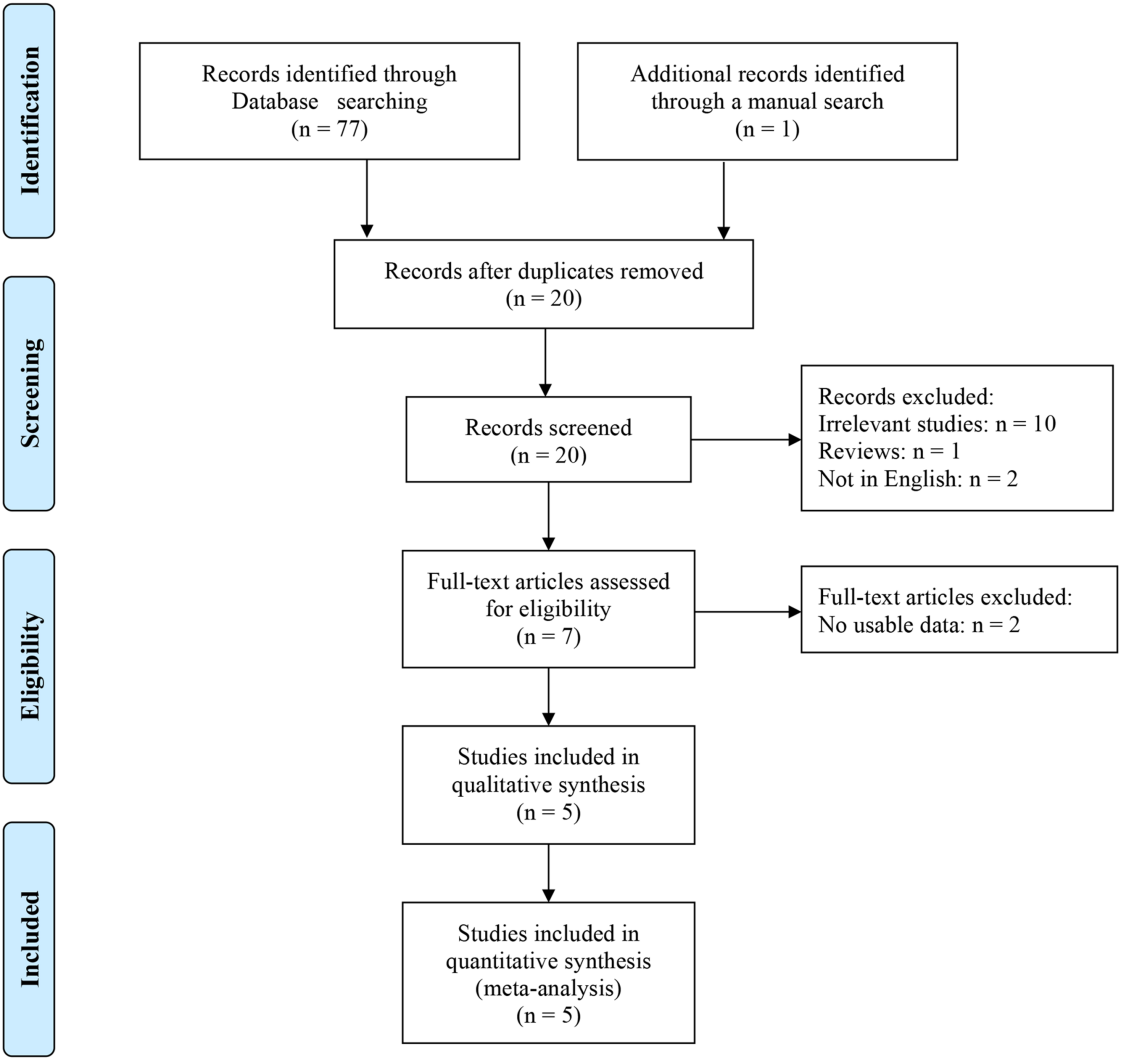
Flow chart of literature search and selection in the meta-analysis.

Details for literature search were shown in Fig 1. Finally, a total of five published documents met our inclusion criteria [20–22, 27–28]. These articles included 523 cases, 564 controls and 87 probands plus 153 relatives reporting the relationship between *DRD2* TaqIA polymorphism and the susceptibility to TS. When stratified by ethnicity, four studies involving Caucasian population contained 372 cases, 381 controls and 87 families, and the only study was respected to Asians including 151 patients and 183 controls. Thus, the meta-analysis by ethnicity was restrained to Caucasians. Five articles had enough information for extracting the numbers of allele A1 and allele A2 in TS patients, controls and probands plus relatives. The selected characteristics of each study were summarized in the Table 1. The present study met the requirements of PRISMA statement (S1 PRISMA Checklist and S2 Text).

**Table 1:** Main characteristics of the studies and populations included in this meta-analysis.

First author	Year	Country(Ethnicity)	Diagnostic criteria	Study Type	Methods	Sample size		Case			Control			P_HWE_
						Cases/Controls	Cases/Parents	A1A1	A1A2	A2A2	A1A1	A1A2	A2A2	
Comings et al. [20]	1991	Non-Hispanic (C)	DSM-III-R	PCC	Probe	147/314	-	9	57	81	7	70	237	0.501
Nothen et al.[21]	1994	Germany (C)	DSM-III-R	FB	Probe	-	61/109	-	-	-	-	-	-	-
Comings et al. [27]	1996	Non-Hispanic (C)	DSM-III-R	PCC	Probe	225/67	-	13	81	131	3	15	49	0.211
Lee et al. [28]	2005	Taiwan (A)	DSM-IV	PCC	RFLP	151/183	-	58	74	19	40	112	31	0.012
Herzberg et al.[22]	2010	Colombia(C)	DSM-IV	FB	RFLP, SNaPshot	-	26/43	-	-	-	-	-	-	-

C: Caucasian; A: Asian; DSM-III-R: Diagnostic and Statistical Manual of Mental Disorders, Revised Third Edition; DSM-IV: Diagnostic and Statistical Manual of Mental Disorders, Fourth Edition; PCC: Population-based case-control study; FB: Family-based; RFLP: Restriction fragment length polymorphism; P_HWE_: P value of Hardy-Weinberg equilibrium.

### Frequency of the A1 allele of the *DRD2* TaqIA polymorphism by ethnicity

Based on the three included case-control studies, the average frequency of the A1 allele of the *DRD2* TaqIA polymorphism was 26.4% in the control group. Caucasians had a lower A1 allele frequency than Asian populations (13.8%). Among healthy controls, the frequency of the A1 allele in the Asians was 52.5% (Table 2). The wide variation of the TaqIA A1-allele frequencies across different ethnicities was very close to that obtained from the data bank of the dbSNP Short Genetic Variations (http://www.ncbi.nlm.nih.gov/snp) (0.19 for CEU and 0.51 for HCB) (S1 Table). In addition, for the two family-based studies, the number of transmitted A1 allele was 74, the number of non-transmitted A1 allele was 106.

**Table 2:** Prevalence of the A1 allele of the *DRD2* gene Taq I A polymorphism.

Populations	Number of studies	Numbers				A1 allele (%)	
		Case	Control	T	NT	Case	Control
Caucasian	4	372	381	74	106	24.5	13.8
Asian	1	151	183	-	**-**	62.9	52.5
Overall	5	523	564	74	106	35.6	26.4

T: Transmitted; NT: Non-transmitted

### *DRD2* TaqIA polymorphism in the meta-analysis

All five studies were combined into the meta-analysis. Table 3 provided the summary of the meta-analysis outcomes regarding to the relationship between the *DRD2* TaqIA polymorphism and TS risk. According to I^2^ index and confidence interval around I^2^, there was no evidence of heterogeneity under the allele model (A1 vs. A2: I^2^ = 38, 95%CI = 0 to 79.0%), homozygous model (A1A1 vs. A2A2: I^2^ = 0, 95%CI = 0 to 89.5%), heterozygous model (A1A2 vs. A2A2: I^2^ = 51, 95%CI = 0 to 85.9%), dominant model (A1A1+A1A2 vs. A2A2: I^2^ = 14, 95%CI = 0 to 55.9%) and recessive model (A1A1 vs A2A2+A1A2: I^2^ = 0, 95%CI = 0 to 89.6%). However, Huedo-Medina et al. suggested that it should be very cautious to make a conclusion for heterogeneity from confidence interval around I^2^ when the number of studies was small [24]. Only five studies were involved in this meta-analysis, so both a fixed-effects model and a random-effects model were utilized for all the five genetic models.

**Table 3:** Meta-analysis of the association between the *DRD2* Taq I A polymorphism and Tourette syndrome.

Polymorphism	Population	Number of studies	Test of association (F)		Test of association (R)		Test of heterogeneity		
			OR	95%CI	OR	95%CI	P value	I^2^	95%CI
A1 versus A2 allele	Overall	5	1.69	1.42–2.00	1.66	1.33–2.08	0.17	38	0–79.0%
	Caucasian	4	1.75	1.43–2.16	1.69	1.25–2.28	0.11	50	0–84.0%
	Asian	1	1.54	1.12–2.10	1.54	1.12–2.10	NA	NA	NA
A1A1 versus A2A2	Overall	3	2.46	1.45–4.20	2.52	1.49–4.26	0.58	0	0–89.5%
	Caucasian	2	2.60	1.14–5.95	2.72	1.21–6.11	0.31	1	0–5.4%
	Asian	1	2.37	1.18–4.76	2.37	1.18–4.76	NA	NA	NA
A1A2 versus A2A2	Overall	3	1.89	1.38–2.58	1.80	1.13–2.87	0.13	51	0–85.9%
	Caucasian	2	2.25	1.57–3.23	2.26	1.58–3.24	0.67	0	0–99.9%
	Asian	1	1.08	0.57–2.05	1.08	0.57–2.05	NA	NA	NA
A1A1+A1A2 versus A2A2	Overall	3	2.05	1.52–2.76	2.04	1.47–2.82	0.31	14	0–55.9%
	Caucasian	2	2.30	1.63–3.24	2.31	1.65–3.26	0.50	0	0–99.8%
	Asian	1	1.42	0.76–2.63	1.42	0.76–2.63	NA	NA	NA
A1A1 versus A1A2+A2A2	Overall	3	2.19	1.44–3.31	2.20	1.46–3.32	0.64	0	0–89.6%
	Caucasian	2	2.07	0.92–4.67	2.12	0.96–4.70	0.35	0	0–53.5%
	Asian	1	2.23	1.38–3.60	2.23	1.38–3.60	NA	NA	NA

OR: Odds ratio; CI:Confidence interval; F:Fixed-effects models; R:Random-effects model; NA:Not available.

**Fig 2:**
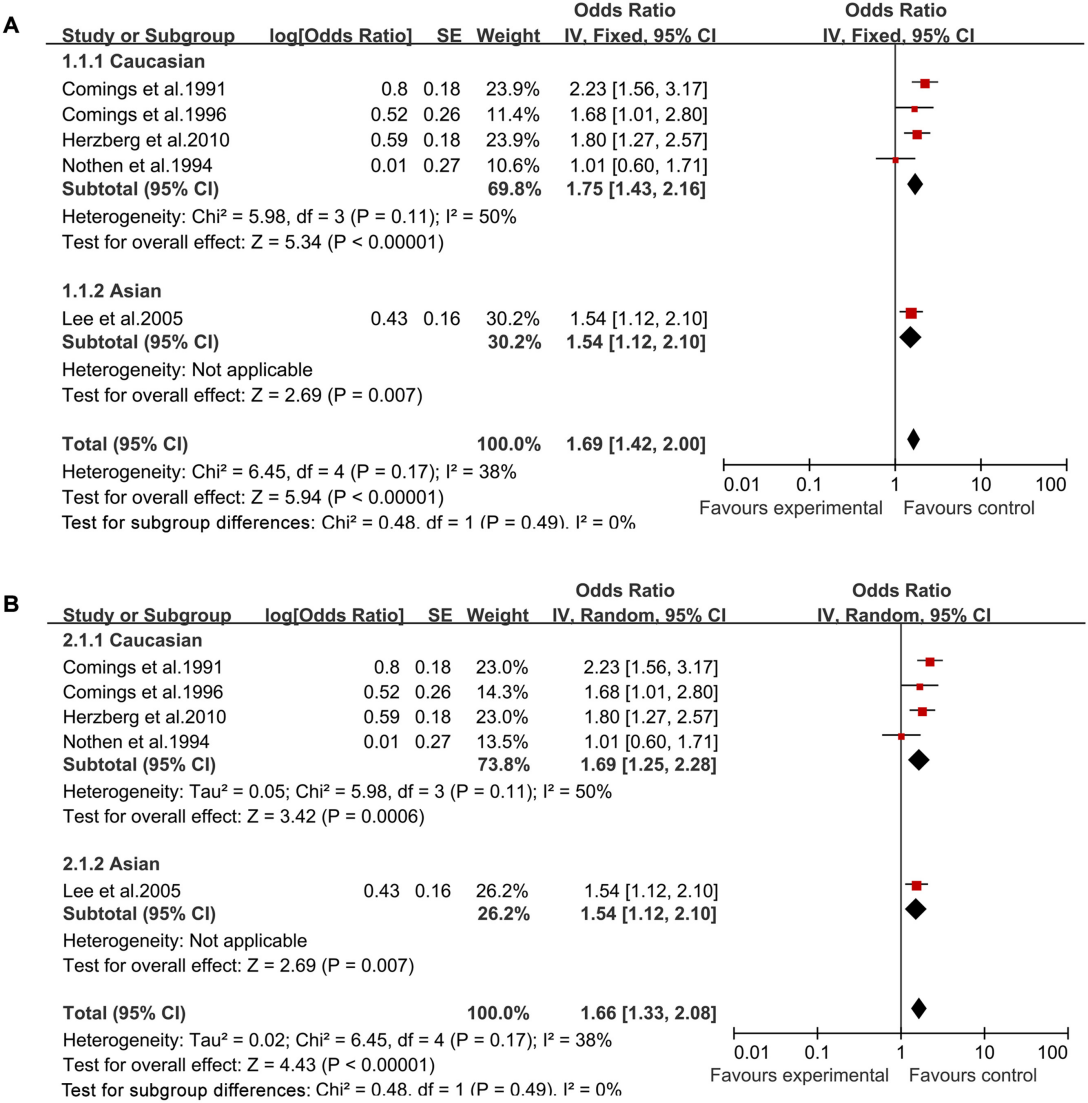
Forest plot of the susceptibility of TS associated with *DRD2* Taq I polymorphism under stratification (A1 vs. A2).

**Fig 3:**
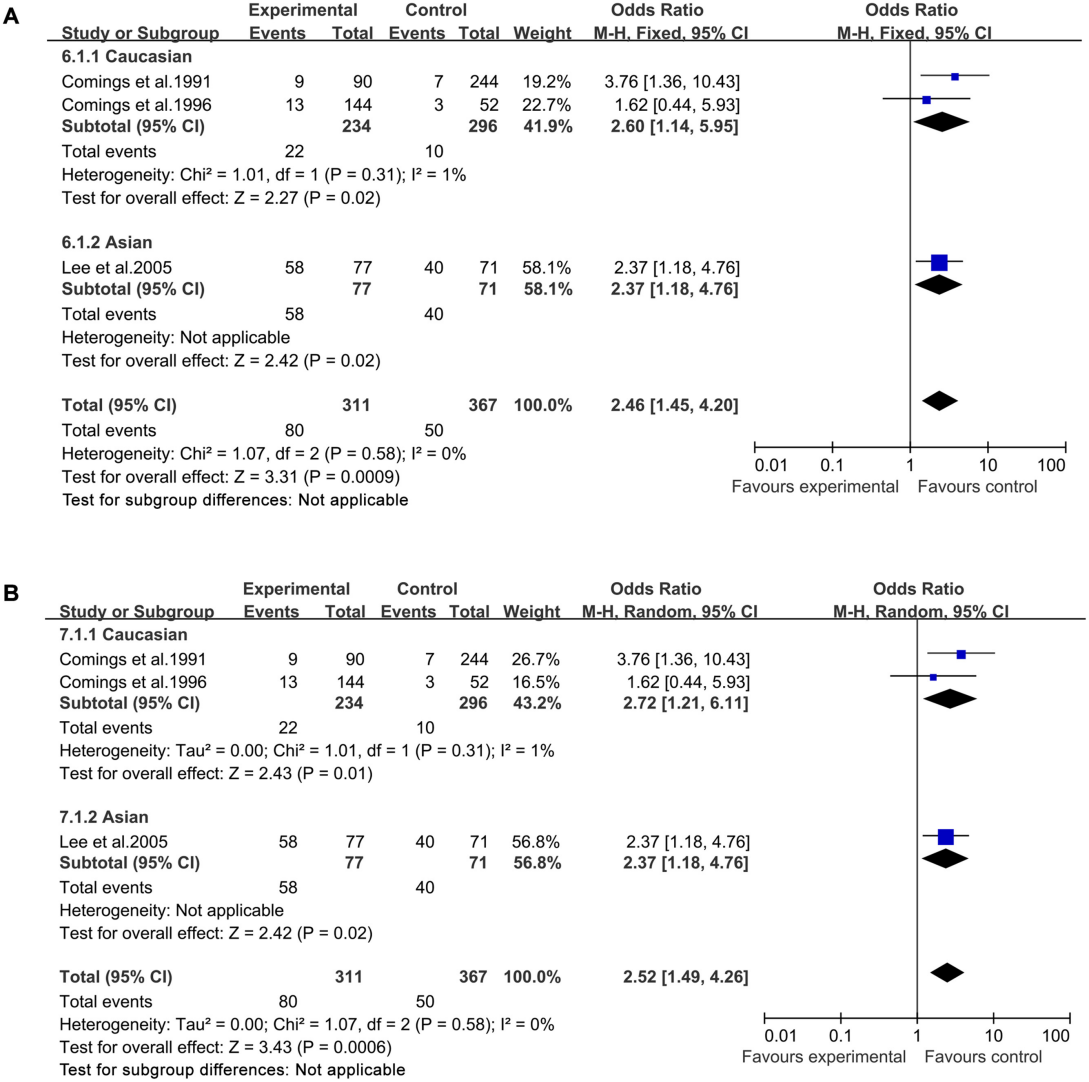
Forest plot of the susceptibility of TS associated with *DRD2* Taq I polymorphism under stratification (A1A1 vs. A2A2).

**Fig 4:**
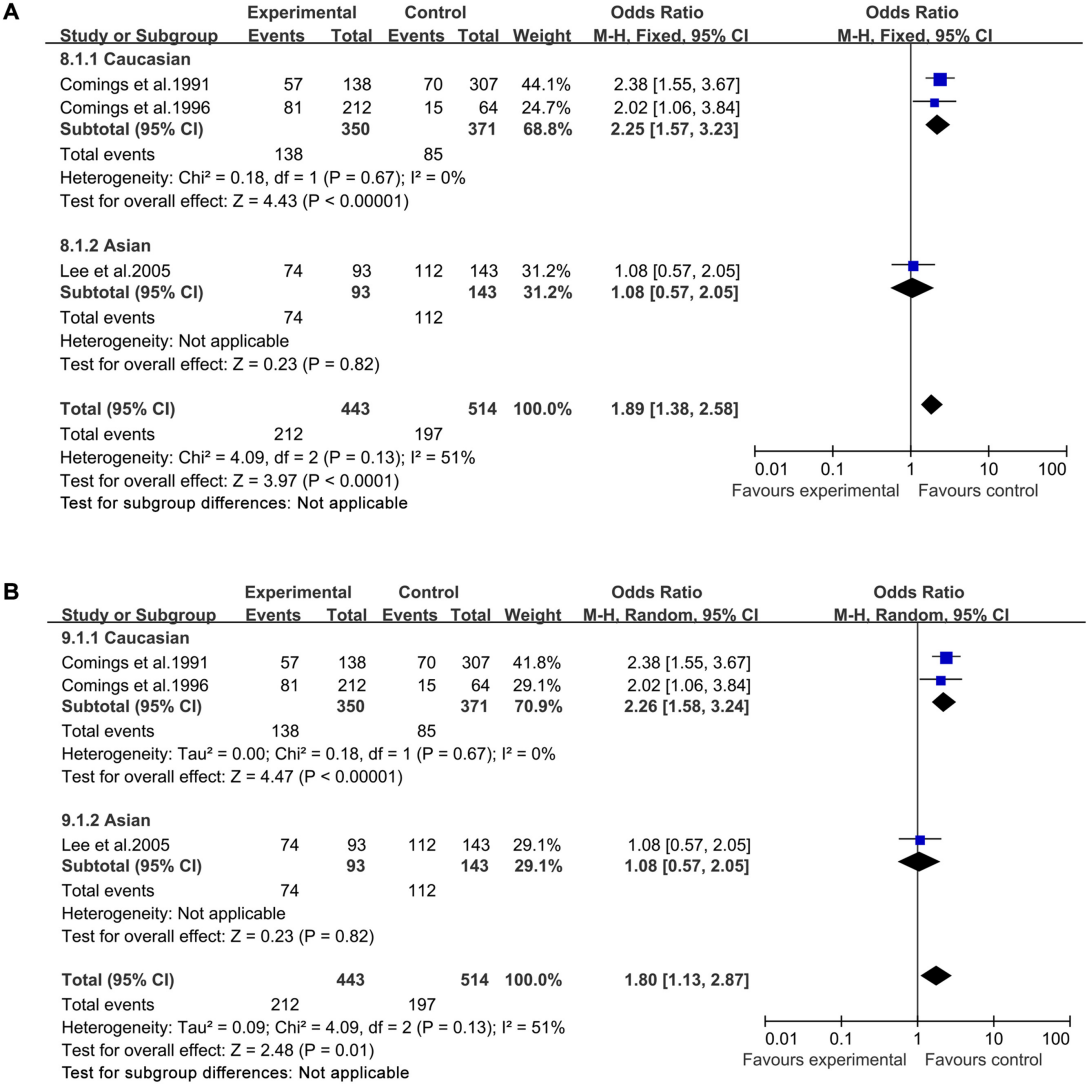
Forest plot of the susceptibility of TS associated with *DRD2* Taq I polymorphism under stratification (A1A2 vs. A2A2).

**Fig 5:**
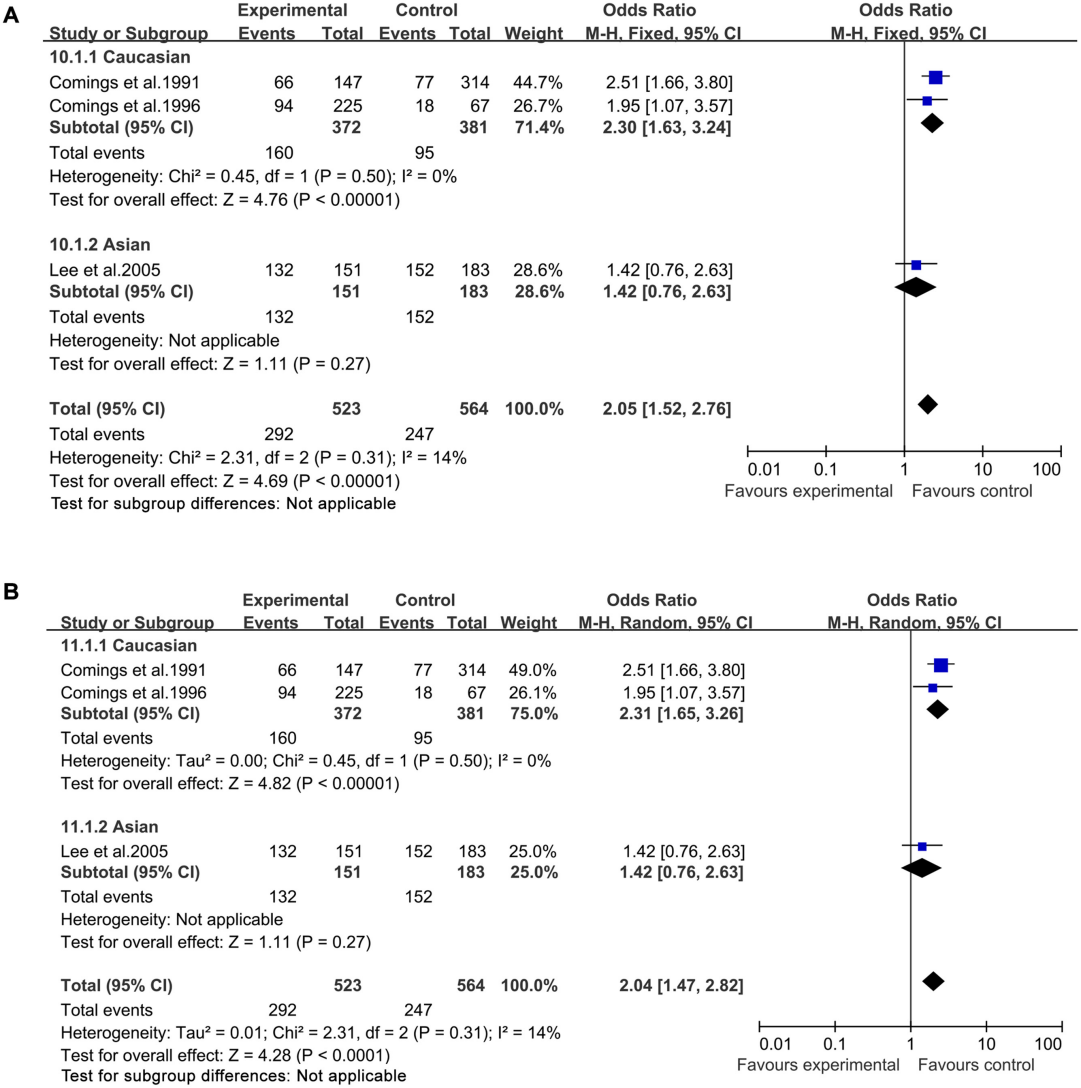
Forest plot of the susceptibility of TS associated with *DRD2* Taq I polymorphism under stratification (A1A1+A1A2 vs. A2A2).

**Fig 6:**
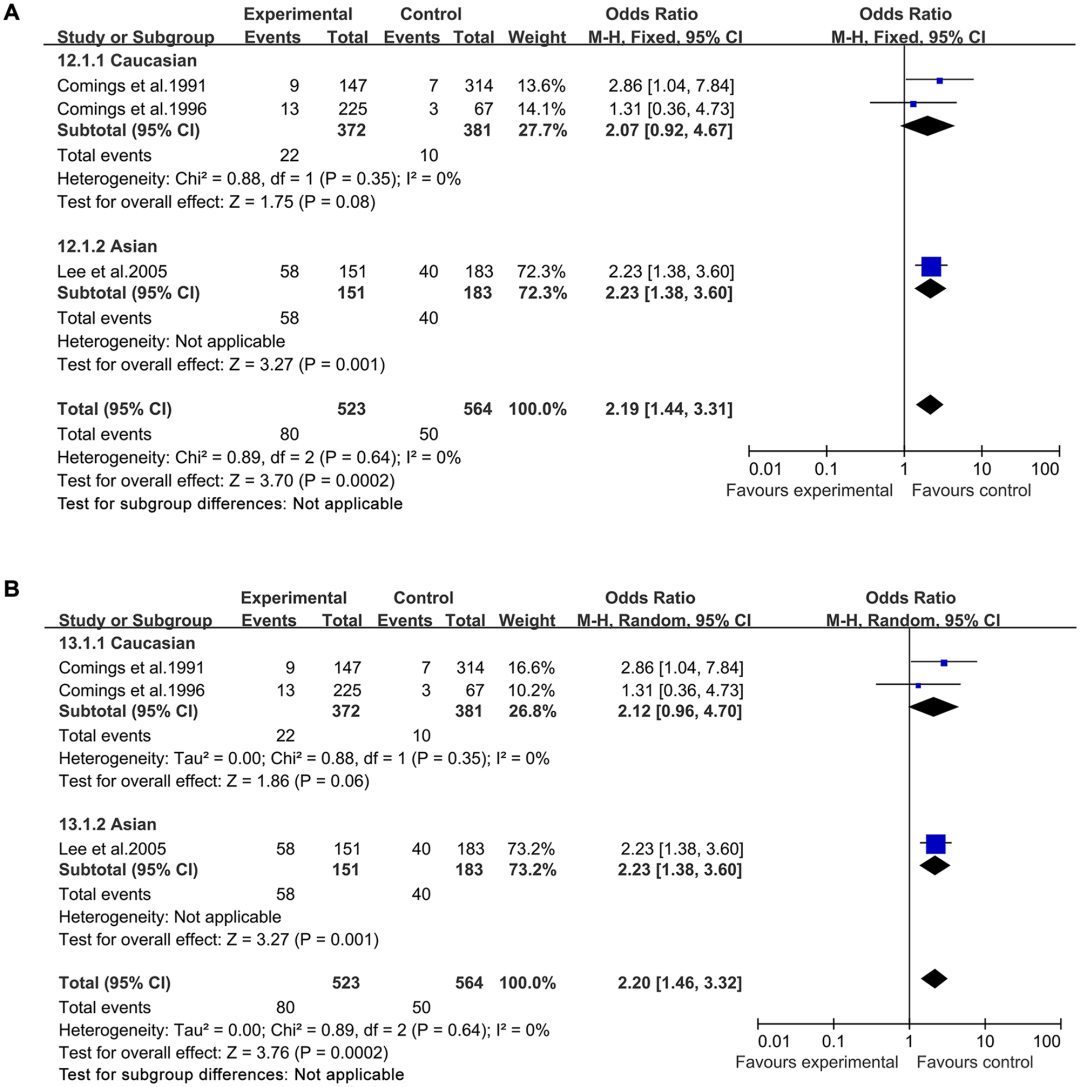
Forest plot of the susceptibility of TS associated with *DRD*2 Taq I polymorphism under stratification (A1A1 vs. A1A2+ A2A2).

In both a fixed-effects model and a random-effects model, significantly increased TS risk was found for A1 allele vs A2 allele (fixed-effects model: OR = 1.69, 95%CI = 1.42–2.00; random-effects model: OR = 1.66, 95%CI = 1.33–2.08, Fig 2), for A1A1 vs A2A2 (fixed-effects model: OR = 2.46, 95%CI = 1.45–4.20; random-effects model: OR = 2.52, 95%CI = 1.49–4.26, Fig 3), for A1A2 vs A2A2 (fixed-effects model: OR = 1.89, 95%CI = 1.38–2.58; random-effects model: OR = 1.80, 95%CI = 1.13–2.87, Fig 4), for A1A1+A1A2 vs A2A2 (fixed-effects model: OR = 2.05, 95%CI = 1.52–2.76; random-effects model: OR = 2.04, 95%CI = 1.47–2.82, Fig 5), and for A1A1 vs A1A2 +A2A2 (fixed-effects model: OR = 2.19, 95%CI = 1.44–3.31; random-effects model: OR = 2.20, 95%CI = 1.46–3.32, Fig 6) (Table 3).

Then stratified analysis was executed to assess the potential ethnic differences. The results from the Caucasian were similar to the overall population in four genetics models including A1 allele vs A2 allele-allele model, A1A1 vs A2A2-homozygous model, A1A2 vs A2A2-heterozygous model and A1A1+A1A2 vs A2A2-dominant model. Significantly increased TS risk was also found for A1 allele vs A2 allele (fixed-effects model: OR = 1.75, 95%CI = 1.43–2.16; random-effects model: OR = 1.69, 95%CI = 1.25–2.28, Fig 2), for A1A1 vs A2A2 (fixed-effects model: OR = 2.60, 95%CI = 1.14–5.95; random-effects model: OR = 2.72, 95%CI = 1.21–6.11, Fig 3), for A1A2 vs A2A2 (fixed-effects model: OR = 2.25, 95%CI = 1.57–3.23; random-effects model: OR = 2.26, 95%CI = 1.58–3.24, Fig 4) and for A1A1+A1A2 vs A2A2 (fixed-effects model: OR = 2.30, 95%CI = 1.63–3.24; random-effects model: OR = 2.31, 95%CI = 1.65–3.26, Fig 5). However, no significantly increased risk was found for A1A1 vs A1A2 +A2A2 (fixed-effects model: OR = 2.07, 95%CI = 0.92–4.67; random-effects model: OR = 2.12, 95%CI = 0.96–4.70, Fig 6) (Table 3). The results from the Asian were similar to the overall population and Caucasian in two genetics models including A1 allele vs A2 allele-allele model (fixed-effects model: OR = 1.54, 95%CI = 1.13–2.10; random-effects model: OR = 1.54, 95%CI = 1.12–2.10, Fig 2) and A1A1 vs A2A2-homozygous model (fixed-effects model: OR = 2.37, 95%CI = 1.18–4.76; random-effects model: OR = 2.37, 95%CI = 1.18–4.76, Fig 3).

### Sensitivity analysis

**Fig 7:**
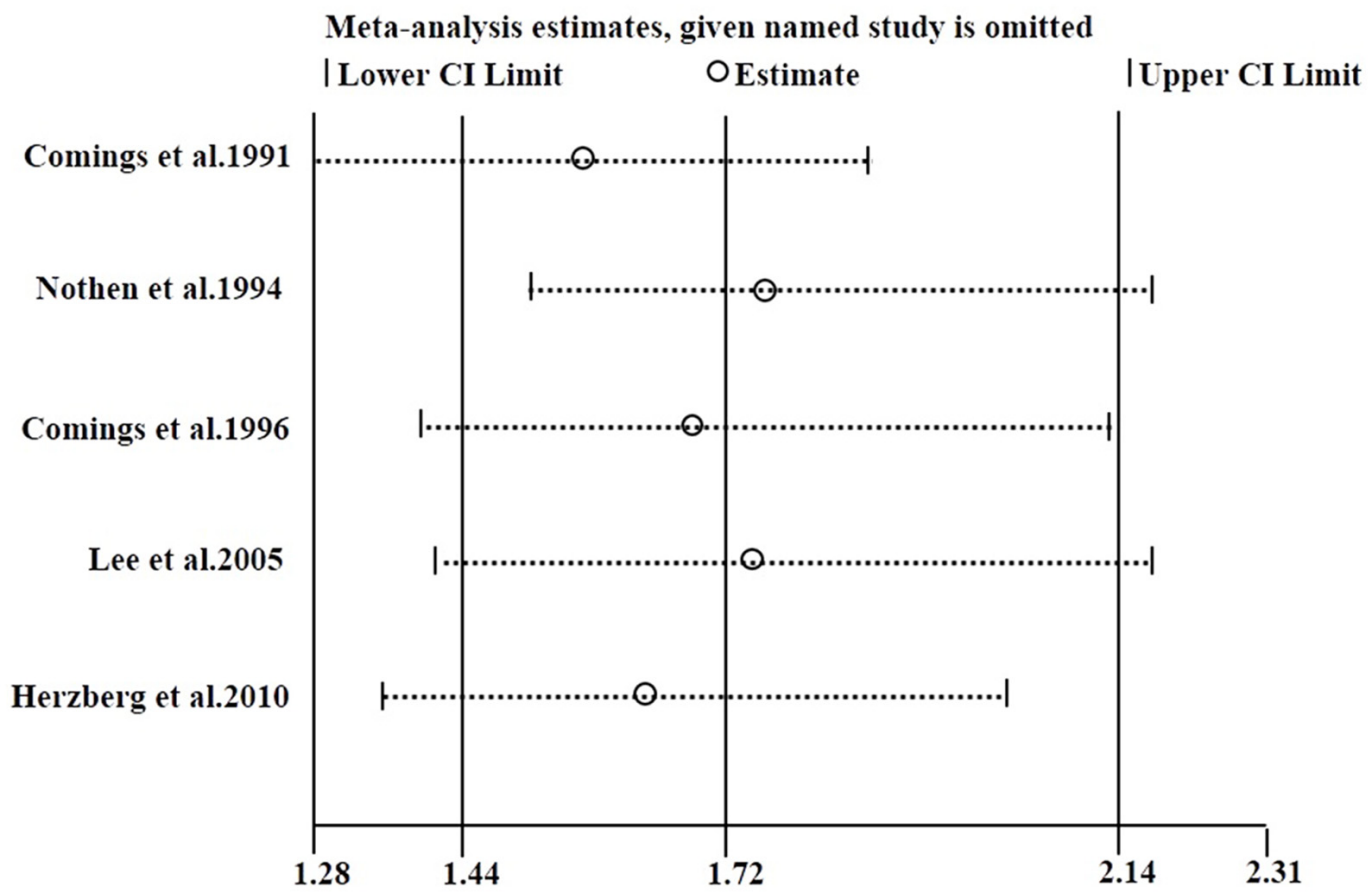
Sensitivity analysis of summary odds ratio coefficients on the relationships between the *DRD2* Taq I polymorphism and the risk of TS (A1 allele vs. A2 allele).

Sensitivity analysis was conducted to assess the influence of each individual study on the pooled ORs. By removing each individual study, the pooled ORs were not altered significantly, which indicated that no individual study significantly affected the pooled results. When one HWE-violating study was excluded, the corresponding pooled ORs were still meaningful, showing the results were relatively credible and stable (OR = 1.75, 95%CI = 1.43–2.16) (Fig 7).

## Discussion

To our knowledge, this is the first comprehensive meta-analysis of studies examining the association of the *DRD2* TaqIA polymorphism with TS up to date. In this meta-analysis, three case-control articles and two family-based articles about the relationship between *DRD2* TaqIA polymorphism and the susceptibility to TS were selected. These studies included 523 cases, 564 controls and 87 families, and four studies regarding Caucasians contained 372 patients, 381 controls and 87 families, and the only article regarding Asian population included 151 cases and 183 controls. Although previous studies failed to confirm the association between *DRD2* TaqIA polymorphism and TS [14, 21–22], the current meta-analysis implied a significant association between *DRD2* TaqIA polymorphism and TS risk under the allele model (A1 vs. A2: fixed-effects model, OR = 1.69; random-effects model: OR = 1.66), heterozygous model (A1A2 vs. A2A2: fixed-effects model, OR = 1.89; random-effects model: OR = 1.80), homozygous model (A1A1 vs. A2A2: fixed-effects model, OR = 2.46; random-effects model: OR = 2.52), dominant model (A1A1+A1A2 vs. A2A2: fixed-effects model, OR = 2.05; random-effects model: OR = 2.04) and recessive model (A1A1 vs A2A2+A1A2; fixed-effects model, OR = 2.19; random-effects model: OR = 2.20). In addition, stratified analysis based on ethnicity was performed. Interestingly, we also found apparent association between *DRD2* TaqIA polymorphism and Caucasians for A1 allele vs. A2 allele, for A1A2 vs. A2A2, for A1A1 vs. A2A2, and for A1A1+A1A2 vs. A2A2. This meta-analysis, based on the updated published data, has further increased sample size and enlarged the statistical power to reflect the accurate effect of *DRD2* TaqIA polymorphism in TS.

Genetic variations in *DRD2* may affect with the metabolism of dopamine and may lead to neurotransmitter dysfunction. In a recent study, the *DRD2* Taq1A polymorphism was reported to be located in a novel kinase gene, designated ankyrin repeat and kinase domain containing 1 protein (ANKK1) (S1 Fig). International HapMap project data showed that *ANKK1* gene containing the Taq1A polymorphism was in linkage disequilibrium (LD) with the *DRD2* gene and the LD had been found to be between the Taq1A polymorphism and C957T polymorphism. In the haplotype analysis, the *DRD2* density tended to be decreased by the C957T C/C genotype in the Taq1A A2/A2 subgroup, but such a trend had not been observed in the A1 allele-containing haplotype [29]. However, evidence from in vivo study revealed that *DRD2* availability was decreased in the carriers of the A1 allele of the Taq1A polymorphism when compared to the non-carriers [30]. Given the above inconsistent results on the association of Taq1A A1 allele with *DRD2* density, it requires further clarification on the role of Taq1A A1 allele in dopamine dysfunction and individual patient’s vulnerability for the development of TS.

The results of the meta-analysis showed allele A1 of Taq1A conferred risk to the susceptibility for TS (fixed-effects model, OR = 1.69; random-effects model: OR = 1.66), which was consistent with the results of previous three individual studies (OR = 2.22; OR = 1.68; OR = 1.54) [20, 27, 28]. Thus, the directions of the effect size for *DRD2* Taq1A in the reported studies and current meta-analysis were same. However, the effect size varied somewhat among the above studies. There may be several reasons for the difference of effect size between the current meta-analysis and previous studies results. The first possible reason relates to the ethnicities differences of the subjects. The differences in the allele frequency of different ethnic populations may be partly responsible for the inconsistent effect size in association studies coming from the different ethnic samples. According to the database of the dbSNP Short Genetic Variations (http://www.ncbi.nlm.nih.gov/snp), allele frequencies of Taq1A were different between Chinese and European population (S1 Table). Therefore, the ethnic differences in allele frequencies may in part explain the inconsistency between the effect sizes of the studies. The second possible reason relates to the heterogeneity of TS. The previous published studies did not show the clinical background data of the patients, such as clinical diagnostic subtypes, biological examination parameters, and family history. Difference in diagnostic subtypes may also contribute to the discrepancies.

Scharf et al. (2013) reported the first genome-wide association study (GWAS) of TS using the samples with European ancestry [31], which showed that no markers achieved a genome-wide threshold of significance (*P* <5×10^−8^). Although our meta-analysis data was consistent with the results of previous association studies on the relationship between the *DRD2* Taq1A polymorphism and TS [20, 27], Taq1A polymorphism was not found to be associated with TS in this GWAS. Interestingly, mutations in the strongest TS candidate gene (*CNTNAP2* and *HDC*) were also not found to confer risk to the susceptibility of TS. The inconsistent results might be due to the clinical phenotype heterogeneity of TS, and allele frequency difference among different ethnic populations. Another possible explanation that the GWAS failed to find significant results for *CNTNAP2* gene and *HDC* gene is that the variants in these two genes are rare. GWAS is underpowered to detect association of rare variants with complex diseases unless the sample size is very large. Although Scharf et al. did not found the significant association between the *DRD2* Taq1A polymorphism and TS, evidence from histological studies and gene expression profile analyses suggested that abnormalities of *DRD2* were involved in the molecular and pathological mechanism of TS [16–18]. In addition, haloperidol, a potent antagonist of DRD2 had been demonstrated to be effective for the treatment of TS [32]. Therefore, it has been supposed that dopamine hyperactivity in the cortex of brain relates to the main pathogenesis of TS [33].

The current meta-analysis has several limitations. First of all, due to the limited availability of the detailed results from the published studies, the sample size of this meta-analysis is still relatively small. We expect that a more accurate evaluation of the cumulative association of *DRD2* with TS could be obtained when more related studies become available. Second, we can’t fully exclude the publication bias. We have not performed a statistical test for the detection of publication bias because these bias tests have very low power in the meta-analysis including only five studies [34]. Third, our results were based on unadjusted estimates. Thus, a more precise analysis could be conducted if individual information was available to permit adjustment. Fourth, the effect of population stratification on our results can’t be avoided, due to three case-control studies using unrelated individuals involved in this meta-analysis. Indeed certain biases arise from population stratification when conducting a case-control study, possibly leading to false discoveries. Therefore, genetically homogeneous subpopulation analysis was applied to reduce the influence of population stratification on the results of association between *DRD2* Taq1A polymorphism and TS. Interestingly, the other three studies with family design considered homogeneous didn’t find a significant association between Taq1A polymorphism and TS [14, 21, 22]. The possible reason for the negative results may relate to the very small sample size of each individual family-based study. In addition, a slight trend toward a higher allele A1 transmission has been reported in Diaz-Anzaldua et al.’s study, which has a bigger sample size and higher statistical power than Nothen’ s study and Herzberg’ s study. Thus, the direction of the effect size of A1 allele in Diaz-Anzaldua et al.’s study is at least consistent with our results. However, we still expect a more accurate evaluation of the association between *DRD2* and TS when more related studies become available.

In a word, as far as we known, this was the first meta-analysis to estimate the relationship between the *DRD2* Taq1A polymorphism and TS risk. In spite of the above limitations, the systematic analysis on the association of *DRD2* Taq1A polymorphism with TS risk has much greater statistical efficiency than any single study. This meta-analysis supports that the Taq1A polymorphism might contribute significantly to the risk of TS, especially for Caucasians. However, in order to better evaluate the association between *DRD2* gene Taq1A polymorphism with the susceptibility to TS, further investigations should be conducted with a larger number of worldwide studies.

## Supporting Information

S1 Fig Human *DRD2* gene structure, *ANNK1* gene structure and location of TaqI SNP. (TIF)

S1 PRISMA Checklist PRISMA 2009 Checklist. (DOC)

S1 Table Allele frequencies of Taq1A in European and Han Chinese population. (XLS)

S1 Text A list of excluded studies. (DOC)

S2 Text Meta-analysis of genetic association studies checklist. (DOC)
