# Supplementary figures and images for: Association between DRD2/ANKK1 TaqIA Polymorphism and Susceptibility with Tourette Syndrome: A Meta-Analysis
Source: PLoS One. 2015 Jun 25;10(6):e0131060. doi: 10.1371/journal.pone.0131060 (PMC4482493; doi:10.1371/journal.pone.0131060)

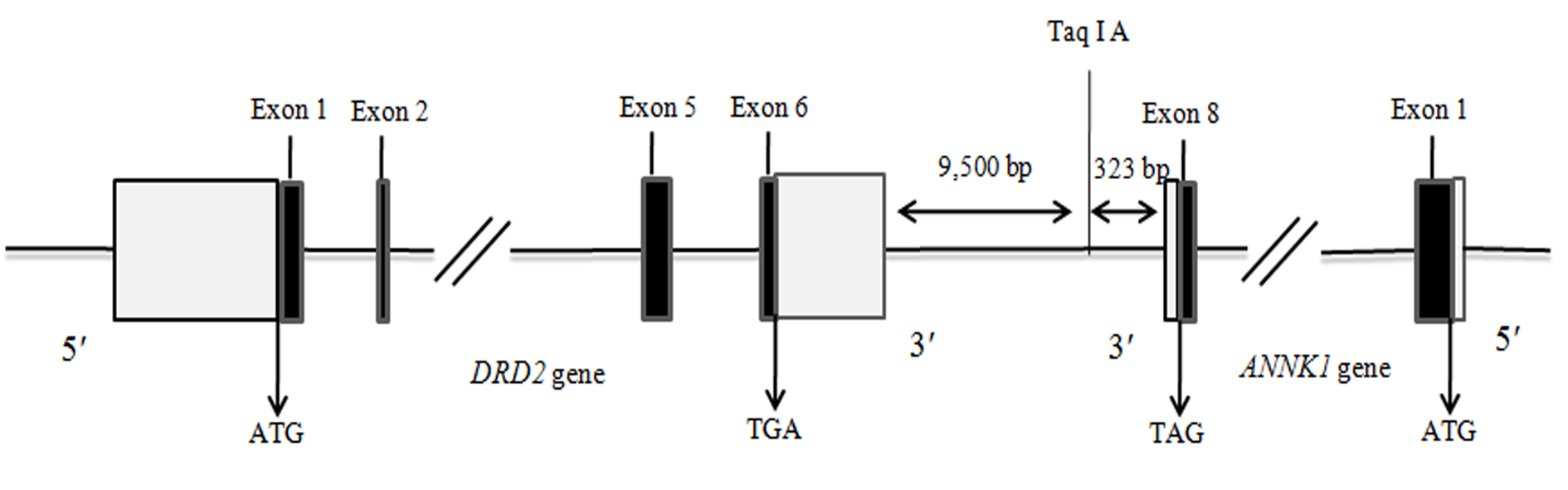

Supplement: S1 Fig — (TIF) [file pone.0131060.s001.tif]
