# Supplementary material for: Association between DRD2/ANKK1 TaqIA Polymorphism and Susceptibility with Tourette Syndrome: A Meta-Analysis
Source: PLoS One. 2015 Jun 25;10(6):e0131060. doi: 10.1371/journal.pone.0131060 (PMC4482493; doi:10.1371/journal.pone.0131060)
Supplement: S1 Text — (DOC) [file pone.0131060.s004.doc]

**A list of excluded studies**

**Irrelevant studies:**

1. Nemoda Z, Szekely A, Sasvari-Szekely M. Psychopathological aspects of dopaminergic gene polymorphisms in adolescence and young adulthood. Neurosci Biobehav Rev. 2011，35(8):1665-1686.
2. Comings DE, Gonzalez N, Wu S, Gade R, Muhleman D, Saucier G, Johnson P, Verde R, Rosenthal RJ, Lesieur HR, Rugle LJ, Miller WB, MacMurray JP. Studies of the 48 bp repeat polymorphism of the DRD4 gene in impulsive, compulsive, addictive behaviors: Tourette syndrome, ADHD, pathological gambling, and substance abuse. Am J Med Genet. 1999, 88(4):358-368.
3. Devor EJ, Dill-Devor RM, Magee HJ. The Bal I and Msp I polymorphisms in the dopamine D3 receptor gene display, linkage disequilibrium with each other but no association with Tourette syndrome. Psychiatr Genet. 1998, 8(2):49-52.
4. Cruz C, Camarena B, King N, Páez F, Sidenberg D, de la Fuente JR, Nicolini H. Increased prevalence of the seven-repeat variant of the dopamine D4 receptor gene in patients with obsessive-compulsive disorder with tics. Neurosci Lett. 1997, 231(1):1-4.
5. Comings DE, Gade R, Wu S, Chiu C, Dietz G, Muhleman D, Saucier G, Ferry L, Rosenthal RJ, Lesieur HR, Rugle LJ, MacMurray P. Studies of the potential role of the dopamine D1 receptor gene in addictive behaviors. Mol Psychiatry. 1997, 2(1):44-56.
6. Comings DE, MacMurray J, Johnson P, Dietz G, Muhleman D. Dopamine D2 receptor gene (DRD2) haplotypes and the defense style questionnaire in substance abuse, Tourette syndrome, and controls. Biol Psychiatry. 1995, 37(11):798-805.
7. Gelernter J, Pakstis AJ, Pauls DL, Kurlan R, Gancher ST, Civelli O, Grandy D, Kidd KK. Gilles de la Tourette syndrome is not linked to D2-dopamine receptor. Arch Gen Psychiatry. 1990, 47(11):1073-1077.
8. Nemoda Z, Szekely A, Sasvari-Szekely M. Psychopathological aspects of dopaminergic gene polymorphisms in adolescence and young adulthood. Neurosci Biobehav Rev. 2011, 35(8):1665-1686.
9. Brett PM, Curtis D, Robertson MM, Gurling HM. The genetic susceptibility to Gilles de la Tourette syndrome in a large multiple affected British kindred: linkage analysis excludes a role for the genes coding for dopamine D1, D2, D3,D4,D5 receptors, dopamine beta hydroxylase, tyrosinase, and tyrosine hydroxylase. Biol Psychiatry. 1995, 37(8):533-540.
10. Barr CL, Wigg KG, Zovko E, Sandor P, Tsui LC. No evidence for a major gene effect of the dopamine D4 receptor gene in the susceptibility to Gilles de la Tourette syndrome in five Canadian families. Am J Med Genet. 1996, 67(3):301-305.

***Reviews:***

1. Comings DE, Blum K. Reward deficiency syndrome: genetic aspects of behavioral disorders. Prog Brain Res. 2000, 126:325-341.

***Not in English:***

1. Huang Y, Liu X, Li T, Guo L, Ma X, Yuan G, Peng R. Transmission disequilibrium test of DRD4 exon III 48bp variant number tandem repeat polymorphism and tic disorder. Zhonghua Yi Xue Yi Chuan Xue Za Zhi. 2002, 19(2):100-103.
2. Kowalska A, Midro AT, Janik P, Gogol A, Służewski W, Rajewski A. Searching for Tourette’s syndrome gene. Part2. Patient’s genome variability. Postepy Hig Med Dosw. 2012, 66:89-95.

***No usable data:***

1. Gelernter J, Pauls DL, Leckman J, Kidd KK, Kurlan R. D2 Dopamine Receptor Alleles Do Not Influence Severity of Tourette's syndrome. Arch Neurol. 1994, 51(4):397-400.
2. Díaz-Anzaldúa A, Joober R, Rivière JB, Dion Y, Lespérance P, Richer F, Chouinard S, Rouleau GA; Montreal Tourette Syndrome Study Group. Tourette syndrome and dopaminergic genes: a family-based association study in the French Canadian founder population. Mol Psychiatry. 2004, 9(3):272-277.
